# Theoretical, Numerical, and Experimental Study on the Identification of Subway Tunnel Structural Damage Based on the Moving Train Dynamic Response

**DOI:** 10.3390/s21217197

**Published:** 2021-10-29

**Authors:** Hongqiao Li, Xiongyao Xie, Yonglai Zhang, Qiang Wang

**Affiliations:** 1 School of Civil Engineering, Tongji University, Shanghai 200092, China; 1410260@tongji.edu.cn (H.L.); zhangyonglai@tongji.edu.cn (Y.Z.); 18310182@tongji.edu.cn (Q.W.); 2 Key Laboratory of Geotechnical and Underground Engineering, Ministry of Education, Tongji University, Shanghai 200092, China

**Keywords:** subway tunnel, moving train, damage identification, wavelet packet energy, acceleration sensor

## Abstract

As an important part of urban rail transit, subway tunnels play an important role in alleviating traffic pressure in mega-cities. Identifying and locating damage to the tunnel structure as early as possible has important practical significance for maintaining the long-term safe operation of subway tunnels. Summarizing the current status and shortcomings of the structural health monitoring of subway tunnels, a very economical and effective monitoring program is proposed, which is to use the train vibration response to identify and locate the damage of the tunnel structure. Firstly, the control equation of vehicle–tunnel coupling vibration is established and its analytical solution is given as the theoretical basis of this paper. Then, a damage index based on the cumulative sum of wavelet packet energy change rate (*TDI_SC_*) is proposed, and its process algorithm is given. Through the joint simulation of VI-Rail and ANSYS, a refined 3D train-tunnel coupled vibration model is established. In this model, different combined conditions of single damage and double damage verify the validity of the damage index. The effectiveness of this damage index was further verified through model tests, and the influence of vehicle speed and load on the algorithm was discussed. Numerical simulation and experimental results show that the *TDI_SC_* can effectively locate the damage of the tunnel structure and has good robustness.

## 1. Introduction

With the continuous expansion of the scale of cities, the size of subways are also increasing and playing an increasingly important role in urban public transportation. Due to construction defects, long service life, environmental erosion, material aging, long-term accumulation of cyclic load of trains, etc., the subways built and operated in the early stages have uneven settlement, segment deformation, segment cracking, segment spalling, segment dislocation, and, behind the wall, problems such as voids and leaking joints [1,2,3]. If these diseases are not monitored and repaired in time, serious subway safety accidents may occur, affecting the travel of millions of people. In 2020, in Shanghai, China, managers needed to maintain 772 km of subway network. However, the average daily maintenance window time is only 3 h and 42 min, while the amount of overhaul construction is increasing at a rate of 10.9% per year. The traditional tunnel structure health monitoring technology mainly includes manual observation and automatic sensor monitoring [4,5,6], and mainly focuses on the surface diseases of the tunnel. Manual observation requires a lot of manpower and is difficult to adapt to the ever-increasing scale of operation and maintenance tasks. Automatic sensor monitoring is fixing sensors on the tunnel structure, which can only be arranged in a small amount in a partial section, as the cost is too high if it is arranged down the whole line of the tunnel.

To solve this dilemma, some mobile monitoring technologies have been introduced into the operation and maintenance of the tunnel. Monitoring technology based on digital cameras and machine vision algorithms are one of the hot spots [7,8,9,10,11,12]. It can analyze the pictures collected by the camera and automatically identify the cracks, water leakage, and other diseases of the tunnel segment. Another tunnel monitoring technology that relies on 3D laser scanning technology has also been very popular recently [13,14,15,16,17]. By analyzing the collected 3D point cloud data, the deformation and convergence of the full section of the tunnel can be monitored, and the accuracy can generally reach the millimeter level. Some higher-precision laser scanners can even monitor the cracks of the tube segments. There are also some studies that combine digital photography with 3D laser scanning technology to study special tunnel inspection vehicles [18,19,20]. The above-mentioned new technologies basically involve installing the monitoring equipment on the mobile test vehicle or manually pushing or motor driving it to achieve mobile monitoring. Compared with the traditional method, the efficiency is greatly improved. However, there are still some shortcomings. First, the moving speed is usually relatively slow. The fastest reported monitoring system has a moving speed of only 30 km/h, and as the moving speed increases, the detection accuracy is greatly reduced. Secondly, due to the huge number of pictures or point cloud data to be processed, the hardware requirements of the computing system are very high, and the equipment is relatively expensive. Moreover, the data post-processing time is long, and it is difficult to feed back the monitoring results in real time. Finally, the above-mentioned new technology can only monitor the apparent diseases of the tunnel and can only monitor during subway outages, so its monitoring efficiency and the diversity of monitoring content still need to be improved. To overcome the shortcomings of static monitoring, some vibration-based damage identification methods have been introduced into the subway tunnel health monitoring research.

Some other scholars have tried to solve this problem by means of dynamic monitoring. Zhou [21] established an analysis model of track–tunnel–soil coupling vibration based on the finite element and transfer matrix method, revealing the special law of vibration wave propagation under the coupling condition of the track system and soil, providing a corresponding theoretical basis for dynamic monitoring of a tunnel structure. Feng et al. [22] analyzed the dynamic response characteristics of tunnel structures and proposed corresponding damage location algorithms for two types of tunnel structures based on the cross-correlation function and transmissibility function theory and verified the applicability of the proposed algorithms under different damage conditions through numerical calculation. Luo et al. [23] proposed a shield tunnel structure damage location method based on a distributed identification strategy. The wavelet packet energy spectrum method is used to analyze the dynamic response signals before and after the tunnel damage under the moving load, and the shield tunnel structure is divided into linearly connected sub-intervals through the mutual information algorithm. Zhang et al. [24] analyzed the acceleration response signal of a shield tunnel structure under environmental excitation. Using the energy spectrum of the acceleration response signal, they proposed two early warning indicators for health monitoring and verified the validity of the proposed warning indicators with the measured data of the Wangzong tunnel in Wuhan Metro Line 3. Although these dynamic monitoring methods can make up for the shortcomings of traditional static monitoring which only consider the apparent diseases of the tunnel, they are still methods that fix the sensors on the structure, and it is also difficult to arrange the entire line in the tunnel.

In the past 10 years, a damage detection method using time-frequency analysis technology has made great progress in the field of civil engineering health monitoring. Sadhu et al. [25] combined the continuous Cauchy wavelet transform and tensor decomposition to track the time-varying characteristics of the modal response of structures, and proposed a new method to detect the progressive damage of structures. Roveri and Carcaterra [26] proposed a new Hilbert–Huang transform-based damage detection method for bridge structures under vehicle load. The technique uses a single point measurement and is able to identify the presence and location of damage along the beam. Das and Saha [27] used a hybrid damage detection technique that combines variational modal decomposition (VMD) and frequency domain decomposition (FDD) to study the effectiveness of damage detection in environments with strong noise pollution. The above-mentioned methods do not rely on the finite element model of the structure, and have strong practical application value, and the future research direction is mainly to combine time-frequency analysis and deep learning technology to further expand their applications [28]. These methods still consider the way of installing sensors at fixed points on a structure.

To overcome the shortcomings of fixing the sensor, Yang et al. tried to install the sensor on a moving train [29,30,31,32]. They proposed a method to extract the natural frequency of the bridge by using the vehicle’s vertical vibration acceleration. Through numerical simulation and field experiments, they verified that the use of EMD, VMD, and band-pass filtering technology can process the vertical acceleration of the vehicle body, extract the first few natural frequencies of the bridge, and judge the health of the bridge. Kong et al. [33] conducted a similar study and proposed a special test vehicle consisting of a tractor and two follow-up trailers to eliminate the adverse effects of driving frequency and road roughness in the process of extracting bridge models. Although their research has made achievements, the research objects are all bridges, without considering the interaction between structure and soil, which obviously cannot be ignored in tunnel structures.

**Figure 1:**
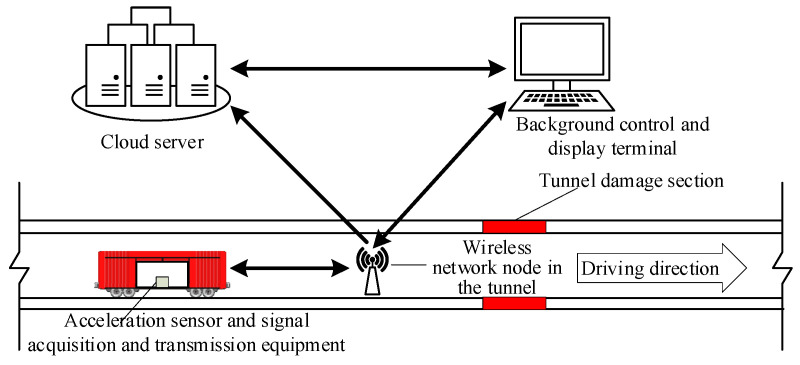
Remote wireless tunnel monitoring scheme based on dynamic response of train in service.

This article also draws on Yang’s idea and proposes the monitoring program shown in Figure 1. The vibration response of the train before and after tunnel failure is collected and analyzed by a vibration sensor installed on the train. The collected original signals and analysis results can be transmitted to the background display terminal and cloud server through the wireless network node in the operation tunnel. The scheme is mature in hardware, but it still lacks the core damage localization algorithm in software. Therefore, the focus of this paper is to propose a damage location algorithm for tunnel structures based on the dynamic response of the train and to verify it through numerical and model tests.

The rest of this paper is organized as follows: in Section 2, the governing equation of vehicle–tunnel coupling vibration is derived and its analytical solution is given as the theoretical basis of this method. In Section 3, the core algorithm of this paper is proposed, that is, the damage localization algorithm based on the cumulative sum of the energy change rate of the wavelet packet. Section 4 and Section 5 verify the algorithm through the numerical model and the experimental model. The results show that this method can locate single damage or multiple damage of the tunnel structure and has good robustness.

## 2. Theoretical Basis

### 2.1. Definition of Tunnel Damage

For the final consequences caused by the diseases, tunnel diseases are classified into two categories [34]. One is the direct degradation of structural stiffness caused by segment cracking, staggered platforms, bolts loosening, and segment material deterioration. The other is the variation of tunnel structure boundary conditions caused by void damage, longitudinal non-uniform settlement, water leakage, and excessive structural deformation. These two kinds of damage eventually lead to the decrease of safety, applicability, and durability of a subway tunnel. Therefore, to simplify the complexity of the theoretical analysis, the damage of a tunnel structure is defined as the degradation of structural stiffness and the variation of boundary conditions.

### 2.2. The Governing Equation of Coupling Vibration between the Train and Tunnel

**Figure 2:**
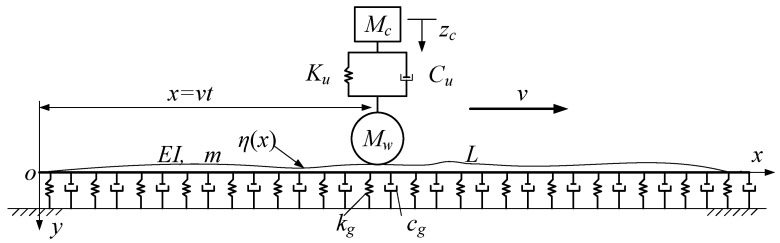
Theoretical calculation model of coupling vibration between train and tunnel.

When a train runs along a track in a subway, the vibration of the track, the track bed, the tunnel, and the soil layer supporting the tunnel is caused by the irregularity of the track and the dynamic load of the train, which in turn acts on the train to make it vibrate. The influence of the vibration of the tunnel is mutual, which is called coupled vibration. The coupled vibration system of the train–track–tunnel–soil system is a very complex structure. To analyze it from the perspective of theory, appropriate simplified assumptions must be made. Herein, the subway tunnel is simplified as a Winkler foundation beam model [34], and the train is considered to have only one carriage and is simplified as a single wheel and mass system attached with a spring and a damper. The overall model is shown in Figure 2. The outer diameter of a subway shield tunnel is generally 6.2 m, and the length between the two stations is generally about 1 km. According to the definition of slenderness ratio, a shield tunnel should belong to a slender beam. Therefore, in Figure 2, the track, track bed, and shield tunnel are considered as a composite beam, which is called a tunnel beam and placed on the Winkler foundation.

Through vertical force analysis of the calculation model in Figure 2, the vibration governing equation of the tunnel beam is

(1) $$EI\frac{\partial^{4}w\left(x,t\right)}{\partial x^{4}}+m\frac{\partial^{2}w\left(x,t\right)}{\partial t^{2}}+c_{g}\frac{\partial w\left(x,t\right)}{\partial t}+k_{g}w\left(x,t\right)=\delta \left(x-vt\right)f\left(t\right),$$

where EI= the equivalent bending stiffness of the tunnel beam, m= the mass of the tunnel beam per meter, $c_{g}=$ the damping coefficient of Winker foundation, $k_{g}=$ the stiffness coefficient of Winker foundation, v= the speed of the train, $w\left(x,t\right)=$ the vertical deflection of the tunnel beam which is the function of time and the coordinates of the beam, $\delta \left(x-vt\right)=$ delta function, and $f\left(t\right)=$ the contact force between the vehicle and the tunnel beam.

The vertical dynamic balance equation of the train includes two parts: the vehicle body and the wheel. In Figure 2, the upper rectangle represents the vehicle body, and the lower circle represents the wheel. By D ‘Alembert’s principle, the dynamic balance equation of the car body and the wheel is

(2) $$M_{c}{\ddot{Z}}_{c}+K_{u}\left(Z_{c}-Z_{w}\right)+C_{u}\left({\dot{Z}}_{c}-{\dot{Z}}_{w}\right)=0,$$

(3) $$M_{w}{\ddot{Z}}_{w}-C_{u}\left({\dot{Z}}_{c}-{\dot{Z}}_{w}\right)-K_{u}\left(Z_{c}-Z_{w}\right)=\left(M_{c}+M_{w}\right)g-f\left(t\right),$$

where $M_{c}=$ the mass of the vehicle body, $M_{w}=$ the mass of the wheel, $K_{u}=$ the stiffness coefficient of the primary spring, $C_{u}=$ the damping coefficient of the primary damper, g= gravitational acceleration, $Z_{c}=$ the vertical vibration displacement of the vehicle body, ${\dot{Z}}_{c}=$ the vertical vibration velocity of the vehicle body, ${\ddot{Z}}_{c}=$ the vertical vibration acceleration of the vehicle body, $Z_{w}=$ the vertical vibration displacement of the wheel, ${\dot{Z}}_{w}=$ the vertical vibration velocity of the wheel, and ${\ddot{Z}}_{w}=$ the vertical vibration acceleration of the wheel. This paper mainly focuses on the vertical vibration of the train and adopts the wheel–rail vertical close-sticking assumption as the basis of the wheel–rail relationship. The formula of the contact relationship between the wheel and the tunnel beam is

(4) $$Z_{w}\left(x,t\right)=\eta \left(x,t\right)+w\left(x,t\right),$$

where $\eta \left(x,t\right)=$ the track irregularity function. In Figure 2, the rail, track bed, and tunnel are regarded as one of the models, so it is equivalent to the irregularity of the top surface of the tunnel beam. The above formulas are the vibration control equations and wheel–rail relationship equations involved in the theoretical model of the vehicle–tunnel coupling vibration. In the following theoretical derivation, to reduce the difficulty of the solution, the track irregularity is not considered for the time being. To obtain a more realistic vehicle body response, the track irregularities will be considered in the numerical model in Section 4.

### 2.3. Analytical Solution of the Governing Equation

According to the vertical unified model theory of the vehicle–track system proposed by Zhai [35], the vibration displacement function of the rail is introduced into the calculation of the tunnel beam in this paper, and its displacement function is

(5) $$w\left(x,t\right)=\sum_{i=1}^{N}Y_{i}\left(x\right)q_{i}\left(t\right),$$

where $Y_{i}\left(x\right)=\sin\left(i\pi x/L\right)$ is the *i*th mode shape function, *L* is the length of the tunnel, $q_{i}\left(t\right)$ is the *i*th modal coordinate of the tunnel beam, and *N* is the total order of calculation.

Regardless of any damping and the irregularity of the track, assume that $c_{g}=0$ and $\eta \left(x,t\right)=0$. By substituting Equations (2)–(5) into Equation (1) and assuming $M_{w}/mL\ll 1,M_{c}/mL\ll 1$, the following equation can easily be obtained:

(6) $${\ddot{q}}_{i}\left(t\right)+\omega_{i}^{2}q_{i}\left(t\right)=\frac{2\left(M_{c}+M_{w}\right)g}{mL}\sin\left(\frac{i\pi vt}{L}\right),$$

where $\omega_{i}=\sqrt{\left(\varpi_{i}^{2}+k_{g}/m\right)}$ and is the *i*th order equivalent circular frequency of a generalized tunnel beam (a tunnel beam on a Winkler foundation) and $\varpi_{i}={\left(i\pi /L\right)}^{2}\sqrt{\left(EI/m\right)}$ is the *i*th order equivalent circular frequency of a tunnel beam. Along with the zero initial conditions for the test vehicle, i.e., $w\left(0,0\right)=0$ and $\dot{w}\left(0,0\right)=0$, Equation (6) can be easily solved, and by substituting $q_{i}\left(t\right)$ back into Equation (5), $w\left(x,t\right)$ can be obtained:

(7) $$w\left(x,t\right)=\sum_{i=1}^{N}\frac{A}{\left(\omega_{i}{}^{2}-B_{i}{}^{2}\right)}\sin\frac{i\pi x}{L}\left(\sin B_{i}t-\frac{B_{i}}{\omega_{i}}\sin\left(\omega_{i}t\right)\right),$$

where $A=2\left(M_{c}+M_{g}\right)/mL$ is a constant, and $B_{i}$ is the pseudo driving frequency of the train. Substituting Equation (7) into Equation (5), $Z_{w}\left(x,t\right)$ can be expressed as follows:

(8) $$\begin{array}{c} Z_{w}\left(x,t\right)\overset{\;x=vt\;}{\to }Z_{w}\left(t\right)=\sum_{i=1}^{N}\frac{A}{\left(\omega_{i}{}^{2}-B_{i}{}^{2}\right)}\left(\sin B_{i}t\right)\left(\sin B_{i}t-\frac{B_{i}}{\omega_{i}}\sin\left(\omega_{i}t\right)\right)\\ \;=\sum_{i=1}^{N}\left(\frac{A}{2\left(\omega_{i}{}^{2}-B_{i}{}^{2}\right)}-\overset{Z_{WV}}{\overbrace{\frac{1}{2}\frac{A}{\left(\omega_{i}{}^{2}-B_{i}{}^{2}\right)}\cos2B_{i}t}}-\overset{Z_{WT}}{\overbrace{\frac{B_{i}}{\omega_{i}}\frac{A}{\left(\omega_{i}{}^{2}-B_{i}{}^{2}\right)}\left(\cos\left(\omega_{i}-B_{i}\right)t-\cos\left(\omega_{i}+B_{i}\right)t\right)}}\right). \end{array}$$

The first term of Equation (8) is the static displacement term, the second term is $Z_{WV}$, and the third term is $Z_{WT}$. The frequency term in $Z_{WV}$ is the pseudo-driving frequency of the train. The frequency term in $Z_{WT}$ is $\omega_{i}-B_{i}$ and $\omega_{i}+B_{i}$. Moreover, $\omega_{i}$ is included in the amplitude term of each component, which means that any factor that causes $\omega_{i}$ to change will cause a change in $Z_{w}$, and thus in ${\dot{Z}}_{w}$ and ${\ddot{Z}}_{w}$.

By putting Equation (8) into Equation (2), dividing both sides by $M_{c}$, ignoring the damping of the car body, i.e., $C_{u}=0$, and letting $\omega_{v}=\sqrt{K_{u}/M_{c}}$, i.e., the circular frequency of the vertical vibration of the vehicle body, the following equation can be obtained:

(9) $${\ddot{Z}}_{c}+\omega_{v}{}^{2}Z_{c}=\overset{p\left(t\right)}{\overbrace{\sum_{i=1}^{N}\frac{A\omega_{v}{}^{2}}{\left(\omega_{i}{}^{2}-B_{i}{}^{2}\right)}\left(\sin B_{i}t\right)\left(\sin B_{i}t-\frac{B_{i}}{\omega_{i}}\sin\left(\omega_{i}t\right)\right)}}.$$

According to the Duhamel integral formula of undamped single-degree-of-freedom system, $Z_{c}$ is calculated as follows:

(10) $$Z_{c}=\frac{1}{\omega_{v}}\int_{0}^{t}p\left(\tau \right)\sin\left(\omega_{v}\left(t-\tau \right)\right)d\tau ,$$

Thus, the expression of $Z_{c}$ can be deduced as follows:

(11) $$Z_{c}=\sum_{i=1}^{N}\left[\overset{Z_{CT}}{\overbrace{C_{1}\cos\left(\omega_{i}-B_{i}\right)t+C_{2}\cos\left(\omega_{i}+B_{i}\right)t}}+\overset{Z_{CC}}{\overbrace{C_{3}\cos\left(\omega_{v}t\right)}}+\overset{Z_{CV}}{\overbrace{C_{4}\cos\left(2B_{i}t\right)}}+C_{5}\right],$$

where $C_{i}\left(i=1\sim 5\right)$ is constant, and their expression is as follows:

(12) $$C_{1}=\frac{B_{i}\omega_{v}{}^{2}A}{2\omega_{i}\left(B_{i}+\omega_{i}-\omega_{v}\right)\left(B_{i}+\omega_{i}+\omega_{v}\right)\left(\omega_{i}{}^{2}-B_{i}{}^{2}\right)},$$

(13) $$C_{2}=\frac{-B_{i}\omega_{v}{}^{2}A}{2\omega_{i}\left(B_{i}+\omega_{i}-\omega_{v}\right)\left(B_{i}+\omega_{i}+\omega_{v}\right)\left(\omega_{i}{}^{2}-B_{i}{}^{2}\right)},$$

(14) $$C_{3}=\frac{-2AB_{i}{}^{2}\left(B_{i}{}^{4}-2B_{i}{}^{2}\omega_{i}{}^{2}+\omega_{i}{}^{4}+2B_{i}{}^{2}\omega_{v}{}^{2}-2\omega_{i}{}^{2}\omega_{v}{}^{2}\right)}{\left(4B_{i}{}^{2}-\omega_{v}{}^{2}\right)\left(B_{i}{}^{2}-{\left(\omega_{i}+\omega_{v}\right)}^{2}\right)\left(B_{i}+\omega_{i}-\omega_{v}\right)\left(B_{i}-\omega_{i}+\omega_{v}\right)\left(\omega_{i}{}^{2}-B_{i}{}^{2}\right)},$$

(15) $$C_{4}=\frac{-\omega_{v}{}^{2}A}{2\left(-2B_{i}+\omega_{v}\right)\left(2B_{i}+\omega_{v}\right)\left(\omega_{i}{}^{2}-B_{i}{}^{2}\right)},$$

(16) $$C_{5}=\frac{A}{2\left(\omega_{i}{}^{2}-B_{i}{}^{2}\right)}.$$

Equation (11) can be divided into four parts: $Z_{CT}$, $Z_{CC}$, $Z_{CV}$ and the constant $C_{5}$. $Z_{CT}$ contains the addition and subtraction terms of the generalized tunnel beam’s equivalent circular frequency and the pseudo driving frequency, i.e., $\omega_{i}-B_{i}$ and $\omega_{i}+B_{i}$. $Z_{CC}$ only have $\omega_{v}$ (circular frequency of the vehicle body) and $Z_{CV}$ only has $B_{i}$ (pseudo-driving frequency of the train). $C_{5}$ can be regarded as the zero-frequency term. In addition, $\omega_{i}$ is included in the amplitude term expression of each component. Therefore when $\omega_{i}$ changes, $Z_{c}$ is bound to change, and so are ${\dot{Z}}_{c}$ and ${\ddot{Z}}_{c}$.

According to the definition of tunnel damage in Section 2.1, the degradation of stiffness and the change of boundary conditions mean the change of parameters such as EI, m, $k_{g}$, and $c_{g}$ leads to the change of $\omega_{i}$. Furthermore, the variation of $\omega_{i}$ leads to changes in the amplitude and frequency of the vibration response of the wheel and the vehicle body. In other words, the time-frequency analysis of the train response can perceive the damage of the tunnel structure through the changes in the time-frequency surface.

## 3. Damage Localization Method

Given the theoretical conclusion deduced in Section 2.3, tunnel damage is reflected in the time-frequency plane change of the train vibration signal. Parameter $\omega_{i}$ is a frequency with order, and the range of frequency distribution is different for different orders. Moreover, the amplitude term corresponding to the order frequency term also contains the frequency order. This means that once the tunnel is damaged, the change of the tunnel’s natural frequency will be reflected in the amplitude term corresponding to each frequency order, which will lead to the change of energy distribution in different frequency bands. Compared with other time-frequency analysis techniques, wavelet packet decomposition can automatically decompose signals to different frequency bands without additional frequency band filters. To extract the variation of train response on the time-frequency plane, this paper introduces the theory of wavelet packet analysis to construct the tunnel damage index and gives the calculation process.

### 3.1. Wavelet Packet Decomposition

In discrete wavelet decomposition [36,37], the signal is usually divided into two parts: high frequency and low frequency. The low frequency part is reserved, and the high frequency part is ignored. With the increase of the number of decomposition layers, the signal concentrates towards the low frequency part, and finally only the approximate signal of the original signal is retained. Wavelet packet decomposition is an optimal decomposition method based on wavelet decomposition. It divides the frequency band into multiple layers, further decomposes the high frequency part that is not analyzed in the discrete wavelet analysis, and adaptively selects the corresponding frequency band according to the characteristics of the analyzed signal to match the signal spectrum, thus improving the time-frequency resolution.

A wavelet packet is usually composed of a linear combination of wavelet functions. With an orthogonal wavelet basis as the parent wavelet, the wavelet packet has the time-frequency property and the orthogonality of the orthogonal wavelet basis. Thus, the wavelet packet is defined as

(17) $$\psi_{j,k}^{i}\left(t\right)=2^{j/2}\psi^{j}\left(2^{j}t-k\right),\;\;\;i=1,2,3\cdots ,$$

where $\psi^{j}$ is an orthogonal wavelet function; *i*, *j*, and *k* are the modulation, scale, and translation coefficients of the wavelet function, respectively; and *t* is the time variable. $\psi_{j,k}^{i}\left(t\right)$ is a normal wavelet package function and is an orthogonal wavelet function. The decomposition recursion formula of the orthogonal wavelet function $\psi^{j}$ is as follows:

(18) $$\left\{\begin{array}{l} \psi^{2j}\left(t\right)=\sqrt{2}\sum_{k=-\infty }^{\infty }h\left(k\right)\psi^{j}\left(2^{j}t-k\right)\\ \psi^{2j+1}\left(t\right)=\sqrt{2}\sum_{k=-\infty }^{\infty }g\left(k\right)\psi^{j}\left(2^{j}t-k\right) \end{array}\right.,$$

where $h\left(k\right)$ and $g\left(k\right)$ are the impact response functions of the low-pass filter and high-pass filter, respectively, corresponding to the wavelet function.

Signal $x\left(t\right)$ is decomposed by the *j*-layer wavelet packet, and $2^{j}$ wavelet packet nodes (decomposition coefficient components) can be obtained:

(19) $$c_{j,k}^{i}\left(t\right)=\int_{-\infty }^{\infty }x\left(t\right)\psi_{j,k}^{i}\left(t\right)dt,$$

Each wavelet packet node can reconstruct a wavelet signal component $x_{j}^{i}\left(t\right)$, which can be expressed as

(20) $$x_{j}^{i}\left(t\right)=\sum_{-\infty }^{\infty }c_{j,k}^{i}\left(t\right)\psi_{j,k}^{i}\left(t\right).$$

Then, the original signal $x\left(t\right)$ can be reconstructed by adding the $2^{j}$ signal component as follows:

(21) $$x\left(t\right)=\sum_{i=1}^{2^{j}}x_{j}^{i}\left(t\right).$$

In addition, if the sampling frequency of the original signal is $f_{s}$, then $x_{j}^{i}\left(t\right)$ (the *j*th layer and the *i*th wavelet signal component) must be distributed in the frequency band range in $\left[i\;f_{s}/2^{j},\left(i+1\right)f_{s}/2^{j}\right]$.

According to the law of conservation of energy, the total energy of signal $E_{S}$ in the time domain is equal to the energy in the time-frequency domain. Then, using the orthogonality of the wavelet basis function, the following equation can be deduced:

(22) $$E_{S}=\int_{-\infty }^{\infty }x^{2}\left(t\right)dt=\sum_{m=1}^{2^{j}}\sum_{n=1}^{2^{j}}\int_{-\infty }^{\infty }x_{j}^{m}\left(t\right)x_{j}^{n}\left(t\right)dt=\int_{-\infty }^{\infty }{\left(x_{j}^{i}\left(t\right)\right)}^{2}dt=\sum_{i=1}^{2^{j}}E_{j}^{i},$$

where $E_{j}^{i}=$ the energy of the original signal’s *j*th layer and the *i*th wavelet signal component. The wavelet packet energy spectrum [38] of $x\left(t\right)$ is defined as a vector:

(23) $$E_{J}=\left[\begin{array}{cccc} E_{j}^{1} & E_{j}^{2} & \cdots & E_{j}^{2^{j}} \end{array}\right],$$

where each element in $E_{J}$ represents the energy value of the original signal in a certain frequency band and their sum is the total energy of the original signal.

For a finite length digital signal $x\left(k\right),k=1,2,3\cdots N$, the energy of the original signal’s *j*th layer and the *i*th wavelet signal component can be obtained by the following expression:

(24) $$E_{j}^{i}=\sum_{k=1}^{N}{\left|c_{j}^{i}\left(k\right)\right|}^{2},$$

where $c_{j}^{i}\left(k\right)$ is the wavelet packet decomposition coefficient at the *j*th layer of the signal $x\left(k\right)$ for the *i*th node at time *k*.

### 3.2. The Definition and Algorithm Flow of the Damage Index

The wavelet packet energy change rate $E_{j,C}^{i}$ can be defined as

(25) $$E_{j,C}^{i}=\frac{\left|E_{j,D}^{i}-E_{j,H}^{i}\right|}{E_{j,H}^{i}},$$

where $E_{j,H}^{i}$ and $E_{j,D}^{i}$ are the energy of the signal’s *j*th layer and the *i*th wavelet signal component in the health state and the damage state, respectively. The rate of change of the wavelet packet energy is a dimensionless quantity. The cumulative sum of the wavelet packet energy change rate $E_{j,SC}$ can be obtained:

(26) $$E_{j,SC}=\sum_{i=1}^{2^{j}}E_{j,C}^{i}.$$

Assuming the sample is under the same conditions, the vibration of the train before and after the tunnel damage is obtained. The sampled signals are divided into equal length intervals and windowed. Then, the cumulative sum of the wavelet packet energy change rate $E_{j,SC}$ corresponding to each window signal is calculated. Due to the theoretical conclusion, the vibration response of the train is changed due to the tunnel damage. $E_{j,SC}$, calculated by the window corresponding to the damage position interval, must be larger than those calculated by the non-damage position interval. Therefore, $E_{j,SC}$ can be used to identify the damage of the tunnel structure. The damage index of a tunnel based on the train’s dynamic response can be defined as *TDI_SC_*, and its expression is

(27) $$TDI_{SC}=\bar{E_{j,SC}}=\sum_{i=1}^{2^{j}}\frac{\left|\bar{E_{j,D}^{i}}-\bar{E_{j,H}^{i}}\right|}{\bar{E_{j,H}^{i}}},$$

where ‘$\bar{}$’ represents the average of the energy of multiple signals. To adapt to multiple measurement data in practical engineering, the average value is obtained to improve its robustness and anti-noise performance.

**Figure 3:**
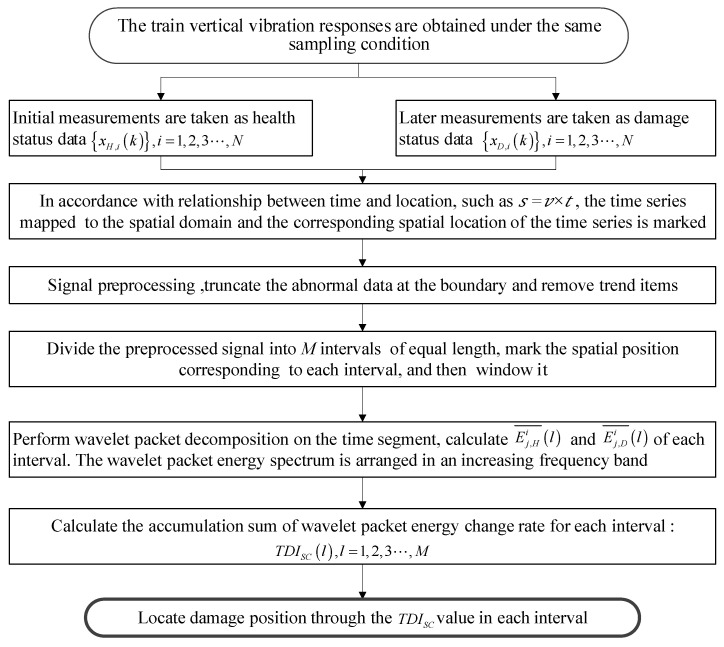
The damage index algorithm flow chart by accumulation sum of wavelet packet energy change.

The specific damage localization algorithm is shown in Figure 3. The input of the process is the train’s dynamic response signal collected under the same conditions in different periods, and the output is the *TDI_SC_* value of each interval. Comparing the *TDI_SC_* values of each interval can determine which interval has been damaged. In addition, usually the frequency of each subsignal obtained by a wavelet packet is not arranged in order of low to high. The wavelet energy spectrum with increasing frequency can be obtained by inverse analysis according to binary Gray code coding rules. In this way, when calculating *TDI_SC_*, the band selection calculation can be carried out according to the analysis of the influence of noise in different frequency bands on the damage location effect, and the frequency bands with large noise influence can be deleted. Thus, the sensitivity and robustness of the damage index *TDI_SC_* to the structural damage will be better.

## 4. Numerical Study and Validation

### 4.1. Verification of 3D Damage Model

In this section, the 3D model is adopted to verify the effectiveness of the *TDI_SC_* algorithm. The 3D model of vehicle–tunnel coupling vibration was established by co-simulation of VI-Rail and ANSYS.

**Figure 4:**
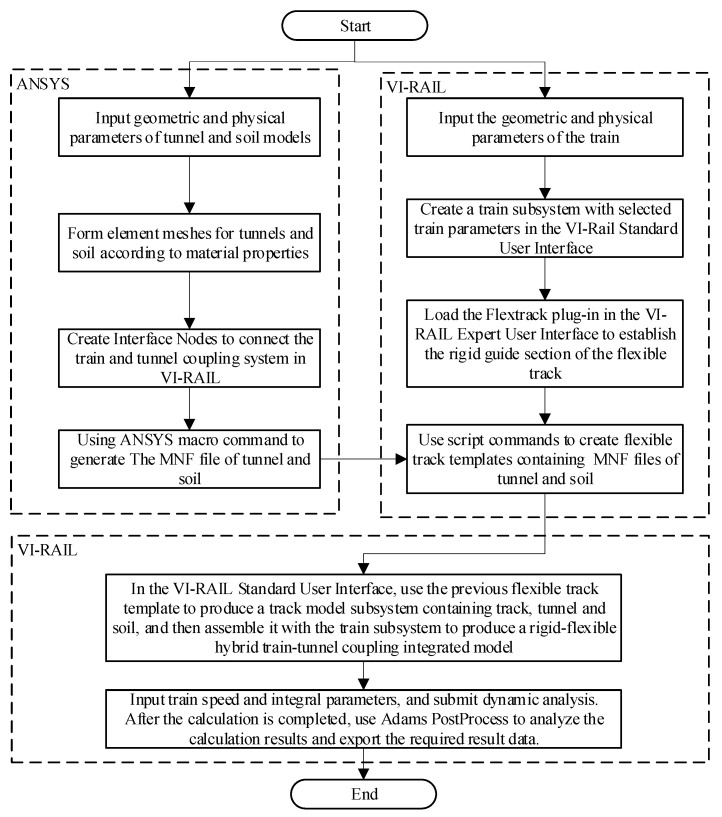
VI-RAIL and ANSYS co-simulation of vehicle–tunnel coupling 3D model modeling process.

This modeling approach was very novel, so this section introduces the basic process of modeling, as shown in Figure 4. The key step of this process is to generate flexible track templates in the VI-RAIL expert user interface. It is necessary to use ANSYS to generate the modal neutral file (MNF) of the tunnel and the soil and select and eliminate some non-essential modalities according to the calculation frequency. The calculations generated by MNF can only be considered in the linear range, so soil and tunnel models can only consider linear constitutive relations. The input parameters include physical geometric parameters such as the length and width of the soil model, the inner and outer radius of the tunnel, the elastic modulus of the tunnel and the soil, and the Poisson’s ratio.

**Figure 5:**
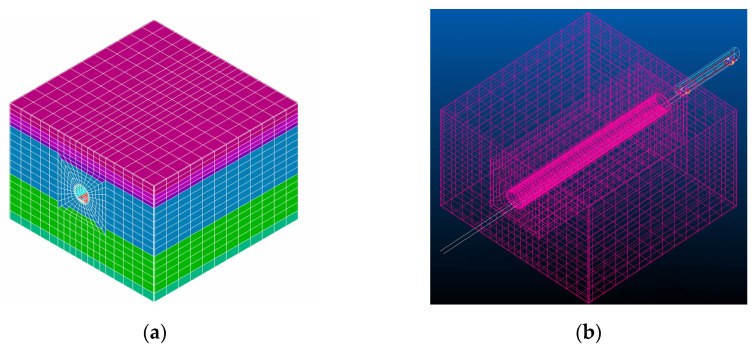
3D vehicle tunnel coupling model: (**a**) ANSYS model of the tunnel and soil and (**b**) vehicle tunnel coupling model in VI-RAIL.

To balance the efficiency of calculation and the scale of the model, only one carriage is considered in the calculation, and its parameters are selected according to the Shanghai Metro Type A train [39]. The parameters of the tunnel model are shown in Table 1. The soil physical parameters in the depth range of the tunnel model were obtained from a typical soft soil layer in Shanghai, as shown in Table 2. Uniform viscoelastic boundary conditions are applied to the four sides of the soil body. According to the principle of dense middle and sparse boundary, the tunnel structure and soil are divided by SOLID45 elements. The boundary is divided by equivalent uniform viscoelastic elements with a thickness of 0.3 m to eliminate the influence of boundary reflection waves. The ANSYS model of the tunnel is shown in Figure 5a, and the overall model of vehicle and tunnel coupling in VI-RAIL is shown in Figure 5b.

**Figure 6:**
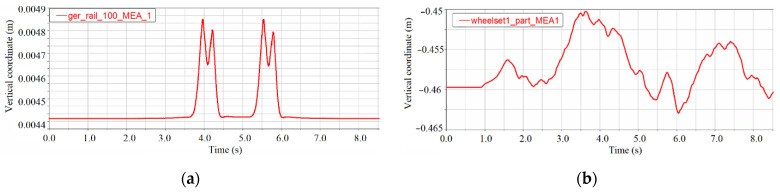
Vibration displacement of the measuring point under the healthy condition of the tunnel: (**a**) vertical vibration displacement of left rail mid-span and (**b**) vertical vibration displacement of the front wheel.

The train speed is 10 m/s, which is 36 km/h, and the sampling frequency is 1000 Hz. It takes 8.947 s for the train to start from the rigid guidance section until the rear wheels leave the tunnel and enter the rigid guidance section. The random irregularity is still considered as an American sixth-grade track spectrum. The vertical vibration displacement of the rail mid-span and the front wheel in a healthy state is shown in Figure 6. The displacement curve in Figure 6a,b is basically similar to those in reference [40], indicating that the 3D simulation process is basically correct.

### 4.2. Verification of the Algorithm

#### 4.2.1. Single Damage Situation

This section verifies the validity of the *TDISC* index for a single damage based on the verification of the correct vehicle–tunnel coupling model in Section 4.1. In the theoretical derivation, any kind of vibration response from a train can reflect a change of tunnel damage. However, in engineering practice, accelerometers are commonly used to collect the vibration response of the structure. Therefore, the vertical acceleration of the center of mass of the front wheelset shaft before and after tunnel damage is used to calculate the *TDI_SC_* index in this numerical simulation verification. In the absence of prior experience, the speed of the train is used as the basis for dividing the positioning interval. In this section, the speed of the train is 10 m/s, so the width of each positioning interval is 10 m, and the tunnel model is divided into six positioning intervals. Generally, a tunnel interval is 1 km, and 10 m is taken as a positioning interval, so the positioning accuracy is appropriate. When calculating the damage index *TDI_SC_*, the Blackman window function is selected as the partition window function, symlet is selected as the wavelet basis function, and the number of decomposition layers is four.

**Figure 7:**
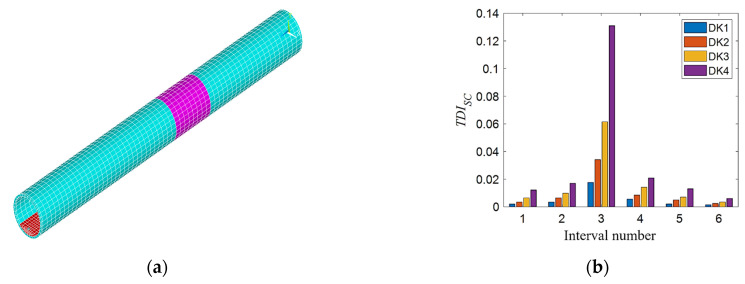
*TDI_SC_* index verification for different degrees of single stiffness damage: (**a**) single stiffness damage tunnel model in the third interval and (**b**) *TDI_SC_* at different degrees of single stiffness damage.

**Figure 8:**
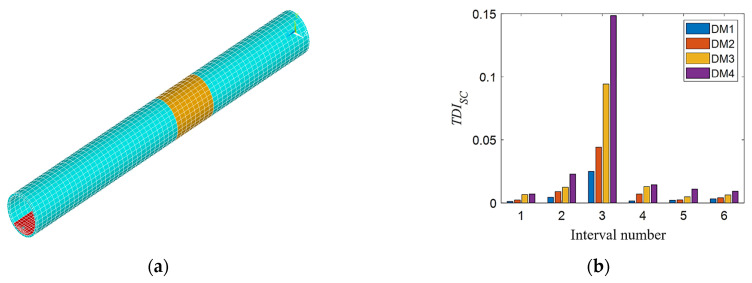
*TDI_SC_* index verification for different degrees of single additional mass damage: (**a**) single additional mass damage tunnel model in the third interval and (**b**) *TDI_SC_* at different degrees of single additional mass damage.

**Figure 9:**
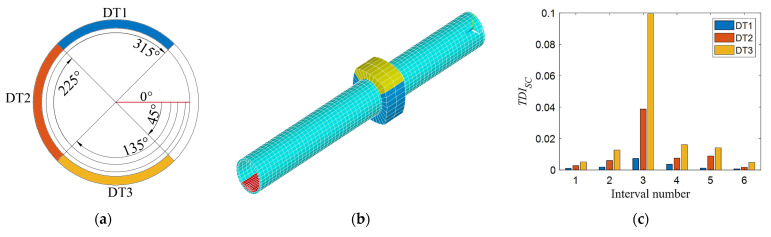
*TDI_SC_* index verification for single void damage in different directions: (**a**) schematic diagram of three different angles of voids on the cross section of the tunnel; (**b**) single void damage tunnel model in the third interval; and (**c**) *TDI_SC_* at different directions of single void damage.

First considering the single stiffness damage, the stiffness damage is simulated by reducing the elastic modulus of the element in the tunnel finite element model. The elastic modulus of the tunnel element at 21~29 m (purple area, as shown in Figure 7a) is reduced by 3%, 6%, 9%, and 12%, respectively, which are four level stiffness damage states. Secondly, considering single additional mass damage, the density of the tunnel unit at 21~29 m (orange-yellow area, as shown in Figure 8a) is increased by 3%, 6%, 9%, and 12%, respectively, which are four additional mass damage states. Finally, the condition of single void damage is considered. The elastic modulus and density of the soil element in the outer ring adjacent to the tunnel are set to near zero to simulate the void damage. The void damage is set at 22.5~27.5 m, and three different angle ranges of void damage are considered in the tunnel cross section as shown in Figure 9a. The blue, red, and yellow areas represent the void damage at 12 o’clock, 9 o’clock, and 6 o’clock, respectively, and their corresponding legend labels are DT1, DT2, and DT3. Figure 9b is a schematic diagram of the tunnel cavity damage at the 12 o’clock direction, in which the yellow-green area represents the void damage, and the blue area represents the normal soil elements. The three void areas are all arc-shaped, with a width of 5 m, a height of about 2 m, and a length of about 11 m.

Figure 7b, Figure 8b, and Figure 9c show the calculated *TDI_SC_* values of each interval under the above three working conditions. As shown in Figure 7b, DK1 to DK4 represent the 1st to 4th level stiffness damage conditions. From the histogram of the same color, the *TDI_SC_* value of the third interval is the largest, which shows that the *TDI_SC_* index can basically locate the damage to the third interval correctly. Affected by track irregularities, there are still some small values on both sides except for the third section, and the interference value decreases as the distance from the third section of the damage location is farther, not affecting the judgment of damage location. As the degree of damage increases, the value of *TDI_SC_* in the third interval also increases. As shown in Figure 8b, DM1 to DM4 represent the 1st to 4th level additional mass damage. For the different degrees of single additional mass damage, the results have similar rules as the stiffness damage. In Figure 9c, obviously, the void damage at three different locations can be accurately located in the third interval by the *TID_SC_* index. Although there are void damages of the same size, the results are different when they are distributed in different locations. The *TDI_SC_* value calculated for the void damage directly under the tunnel at 6 o’clock is the largest, followed by 9 o’clock, and the smallest at 12 o’clock, indicating that the void damage directly below the tunnel has the greatest impact on train vibration.

#### 4.2.2. Two Damages Situation

**Figure 10:**
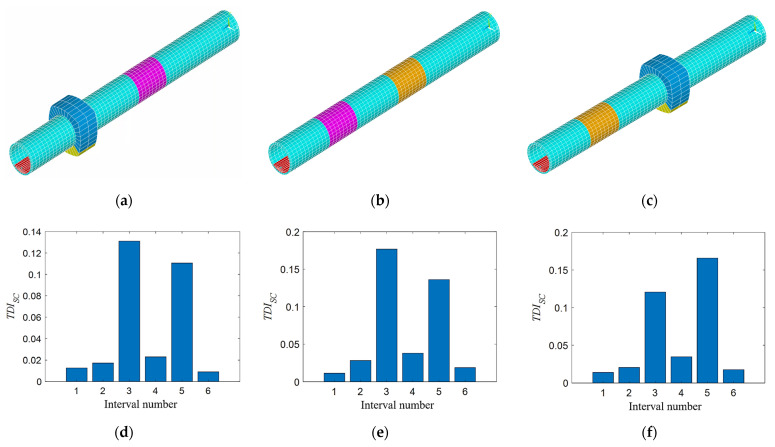
*TDI_SC_* index verification for different types of two damage combinations: (**a**) stiffness and void damage; (**b**) additional mass and void damage; (**c**) void and additional mass damage; (**d**) *TDI_SC_* at stiffness and void damage; (**e**) *TDI_SC_* at additional mass and void damage; and (**f**) *TDI_SC_* at additional mass and void damage.

Three damage conditions are set up in this section, all of which are a combination of different types of damage in the third and fifth intervals. For damage condition 1, in the tunnel model, the elastic modulus of the damaged tunnel element is reduced by 6% at 21~29 m (purple area). The void damage is set at 42.5~47.5 m and the angle range is the same as DT3 in Section 4.2.1, as shown in Figure 10a. Here, the void damage can be realized by setting the elastic modulus and density of the void soil element to a very small value that is close to zero. For damage condition 2, the density of the tunnel element at 21~29 m (orange-yellow area) is increased by 12% as additional mass damage, and the elastic modulus of the damaged tunnel element is reduced by 12% at 41~49 m (purple area), as shown in Figure 10b. For damage condition 3, the void damage is set at 22.5~27.5 m, the angle range is the same as DT3 in Section 4.2.1, and the elastic modulus of the damaged tunnel element is reduced by 12% at 41~49 m (purple area), as shown in Figure 10c. The above three groups of damage condition are in the third and fifth intervals. When calculating the damage index *TDI_SC_*, the parameters are the same as in Section 4.2.1, and the results are shown in Figure 10d–f. As shown, the *TDI_SC_* values of the third and fifth intervals in each figure are the two largest. It shows that the *TDI_SC_* index is accurately positioned to the preset damage interval. This means that the *TDI_SC_* index is also applicable to the multiple tunnel damages condition in the 3D model.

#### 4.2.3. Influence of Different Noise Levels

**Figure 11:**
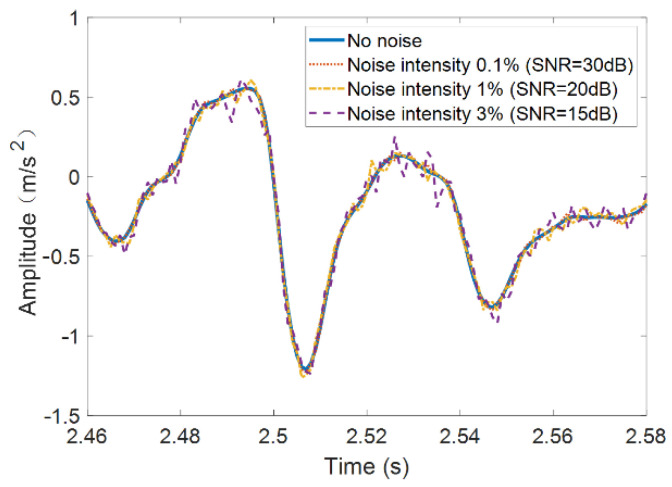
Wheel acceleration in healthy state under different noise levels.

**Figure 12:**
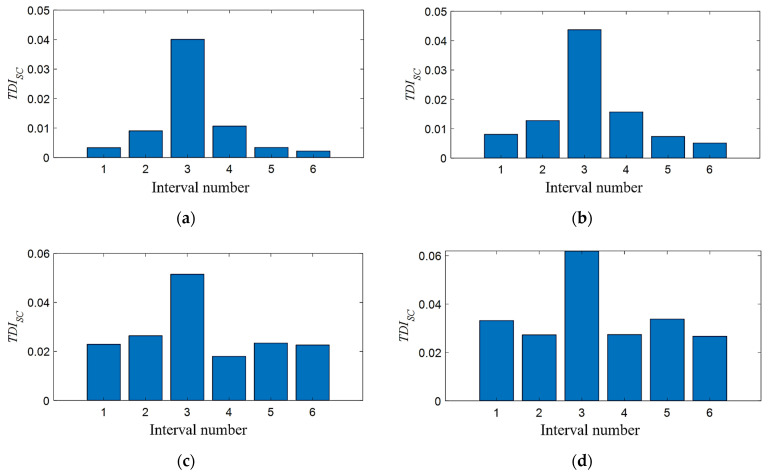
*TDI_SC_* under different noise levels when there is 6% tunnel stiffness damaged in the third interval: (**a**) no noise; (**b**) noise intensity of 0.1% (SNR = 30 dB); (**c**) noise intensity of 1% (SNR = 20 dB); and (**d**) noise intensity of 3% (SNR = 15 dB).

This section studies the damage detection performance of the *TDI_SC_* under various noise levels. The elastic modulus of the tunnel element at 21~29 m is reduced by 12%. Different levels of Gaussian noise are introduced to the vertical acceleration of the center of mass of the front wheelset shaft, then the damage detection procedure is carried out as before. Three different noise-to-signal levels (0.1%, 1%, and 3%) are studied, i.e., corresponding signal-to-noise ratios (SNR) are 30 dB, 20 dB, and 15 dB, as shown in Figure 11. To clearly see the difference between the noise signal and the original signal, only part of the fragments is shown here. When calculating the damage index *TDI_SC_*, the parameters are the same as in Section 4.2.1, and the results are shown in Figure 12.

In the case of the introduction of noise, the *TDI_SC_* index can still accurately identify the preset damage location as shown in Figure 12. Meanwhile, it can be observed that as the noise intensity increases, the *TID_SC_* value of the undamaged area also gradually increases, which means that the interference when locating the damage interval also increases. However, in general, under the preset noise level, the *TDI_SC_* index is still valid, indicating that it has good anti-noise performance.

## 5. Experimental Study and Validation

### 5.1. Establishment of Experimental Model

In this section, model tests are conducted to verify the effectiveness of the *TDI_SC_* algorithm. The similarity ratio between the model and prototype is an important parameter in the model test. Due to the research purposes, the scale model test in this study is preferentially selected to meet the gravity similarity rule, namely, the acceleration similarity ratio between the test model and the prototype is 1. The geometric similarity ratio and elastic modulus similarity ratio are set as 20, and the density similarity ratio is calculated as 2.78, as shown in Table 3.

**Figure 13:**
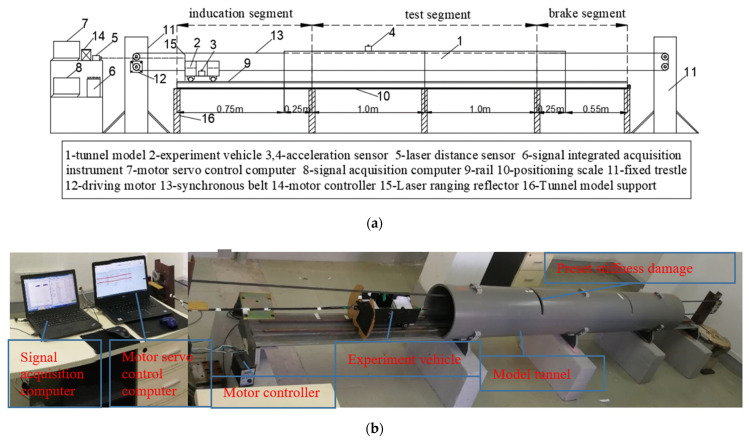
The experimental model: (**a**) the structure diagram of the experimental model and (**b**) the physical drawing of the experimental model.

According to the set elastic modulus similarity ratio, the pipe made of polypropylene material was selected as the tunnel model. The structure diagram of the experimental model is shown in Figure 13. The overall length of the model is 4 m, in which the total length of the tunnel is 2.5 m and the test segment is 2 m.

**Figure 14:**
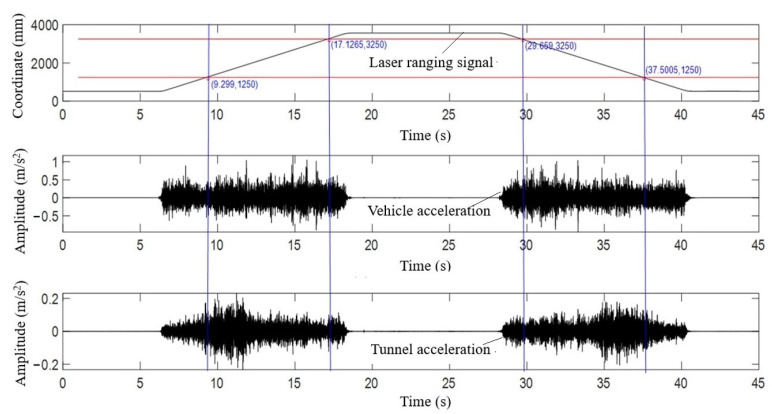
The interception of the original signal in synchronous acquisition.

The test model can be divided into three parts: (1) vehicle–tunnel coupled vibration scale model, (2) vehicle drive control system, and (3) signal acquisition and measurement system. The experiment vehicle is pulled by the rubber synchronous belt, and the reciprocating motion of the car on the track is controlled by the deceleration motor. The experimental vehicle has front and rear axles, which are equivalent to the bogie of a train. The change of the load of the train is simulated by attaching different counterweights. The sensor is installed in the vehicle body, which is equivalent to measuring the vertical acceleration of the train bogie. To align the acceleration data before and after damage in space as much as possible, the synchronous acquisition technology was used to collect the acceleration and laser ranging signals at the same time, and the ruler was used to calibrate the corresponding spatial position of the laser ranging. The two slash segments on the top diagram in Figure 14, i.e., the forward slash and the back slash, respectively, represent the forward and reverse driving segments of the vehicle. The middle and the lower diagram represent the vertical vibration acceleration of the vehicle and the tunnel collected synchronously. In Figure 14, according to the spatial position of laser ranging positioning, the acceleration data for calculation in each group of experiments (the acceleration signal corresponding to the oblique segment of the laser ranging signal intercepted by each group of blue lines) are intercepted.

**Figure 15:**
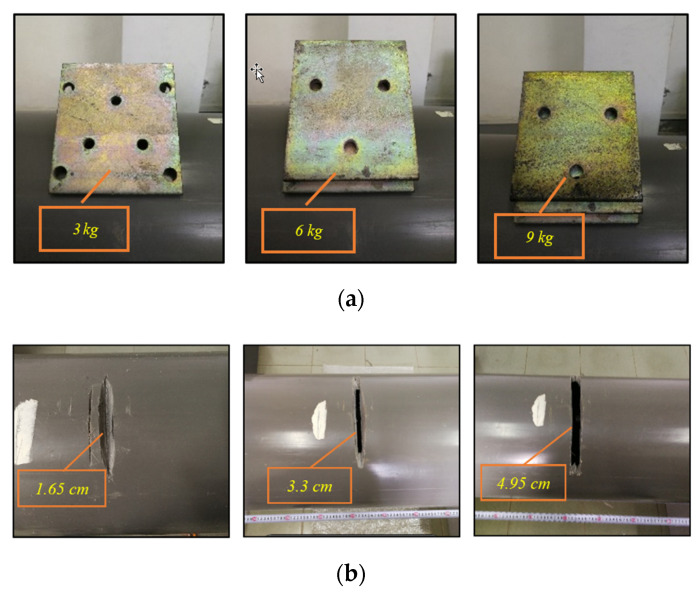
Damage conditions setting: (**a**) additional mass conditions and (**b**) stiffness damage conditions.

The division of tunnel intervals in this section is different from the convention in numerical simulation. The test tunnel section is divided into six positioning intervals. According to the purpose of the experiment, the experiment conditions are set as shown in Table 4. Firstly, the effectiveness of the *TDI_SC_* algorithm for single damage and two damages is verified, and then the influence of vehicle speed and vehicle weight is analyzed. For each group of working conditions, the trolley goes back and forth 10 times, the sampling frequency is 2000 Hz, and each counterweight block in the test vehicle is 0.5 kg. For level 1, 2, and 3 additional mass damage, 3, 6, and 9 kg aluminum alloy mass blocks are added, respectively, in the preset damage interval, as shown in Figure 15a. For level 1, 2, and 3 stiffness damage, the tunnel is cut 1.65 cm, 3.3 cm, and 4.95 cm, respectively, in the preset damage interval, and the corresponding reduced stiffness is 5%, 10%, and 15%, as shown in Figure 15b.

Due to the influence of the model track, the processing accuracy of the trolley, and environmental factors, a large amount of noise is mixed in the measured experimental data. In the calculation process, the input data is first aligned and smoothed to remove noise. The alignment process is to intercept acceleration signals of the same position segment of the car with synchronous position acquisition signals, and further align the intercepted signals with the function “alignsignals(_)” in MATLAB 2017a. To suppress some high-frequency noises and burrs, the five-point cubic smoothing method is used to process the collected acceleration signals. Then, the frequency band is selected according to the frequency band of the tunnel acceleration when calculating the *TDI_SC_*. In this way, the anti-noise ability of the *TDI_SC_* index is improved.

**Figure 16:**
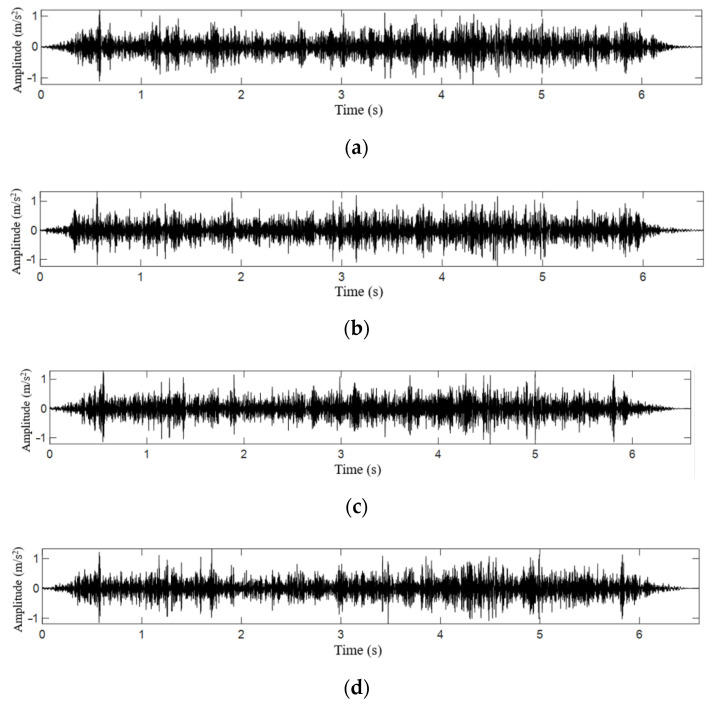
The original signal of vehicle acceleration corresponding to different additional mass damage: (**a**) vehicle acceleration corresponding to health state; (**b**) vehicle acceleration corresponding to Level 1 additional mass damage; (**c**) vehicle acceleration corresponding to Level 2 additional mass damage; and (**d**) vehicle acceleration corresponding to Level 3 additional mass damage.

Figure 16 shows a set of original data of vertical vibration acceleration of the experiment vehicle. Figure 16a is the corresponding original vehicle acceleration signal in a healthy state. Figure 16b–d corresponds to the additional mass damage of 1, 2, and 3, respectively. A large amount of test noise is mixed into these acceleration time-domain signals, and the data cannot guarantee complete alignment. So, it is impossible to directly make the subtraction to reflect the damage location of the tunnel.

### 5.2. Verification of Single Damage

**Figure 17:**
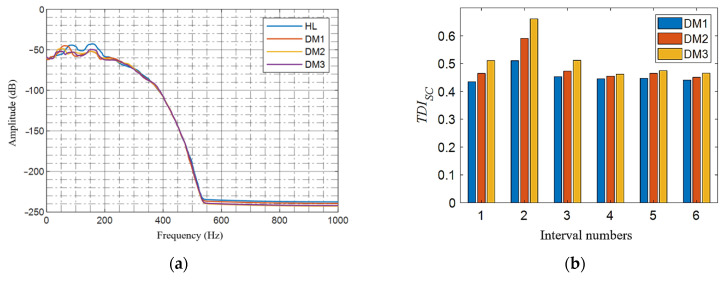
Verification of single additional mass damage in different degrees: (**a**) power spectrum curve of the tunnel acceleration under different degrees of additional mass damage in the second interval and (**b**) *TDI_SC_* at different degrees of additional mass damage in the second interval.

**Figure 18:**
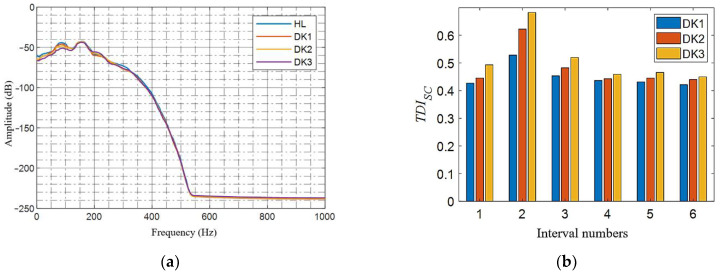
Verification of single stiffness damage in different degrees: (**a**) power spectrum curve of the tunnel acceleration under different degrees of stiffness damage in the second interval and (**b**) *TDI_SC_* at different degrees of stiffness damage in the second interval.

First, the effectiveness of the *TDI_SC_* algorithm for single damage is verified. The damage of single added mass and single stiffness of different degrees are calculated, respectively, corresponding to the serial numbers 1 to 7 in Table 4. When calculating the damage index *TDI_SC_*, the Blackman window function is selected as the partition window function, symlet is selected as the wavelet basis function, and the number of decomposition layers is 5. The sampling frequency is 2000 Hz and the number of wavelet decomposition layers is 5, so the bandwidth of each frequency band after decomposition is 31.25 Hz. According to the distribution of the acceleration spectrum on the tunnel wall before and after the tunnel damage, Figure 17a and Figure 18a shows that the acceleration of the tunnel is mainly distributed in the range of [0, 250] Hz, and the energy change caused by the damage is also distributed within this frequency band. Therefore, when calculating *TDI_SC_*, only the energy change in this frequency band is considered, and other frequency bands do not participate in the calculation, which is equivalent to only calculating the cumulative sum of the energy change rates of the wavelet packets in the first six frequency bands. Other working conditions in this section are calculated as follows. The calculation results are shown in Figure 17b and Figure 18b. In the legend, HL, DM1, DM2, and DM3 correspond to tunnel health, level 1 additional mass, level 2 additional mass, and level 3 additional mass damage conditions, respectively. DK1, DK2, and DK3 correspond to tunnel health, level 1 stiffness, level 2 stiffness, and level 3 stiffness damage conditions, respectively. The same color column state corresponds to the same group of working conditions. Compared with the results of numerical simulation (Section 4.2.1), it is obvious that the model test is disturbed by a large amount of noise. From the blue, red, and yellow color histograms, the three color bars in the second interval take the maximum value. This means that the *TDI_SC_* algorithm can correctly locate pre-defined single injury. In addition, with the increase of the damage degree, the *TDI_SC_* value corresponding to the damage interval also increases.

### 5.3. Verification of Two Damages

**Figure 19:**
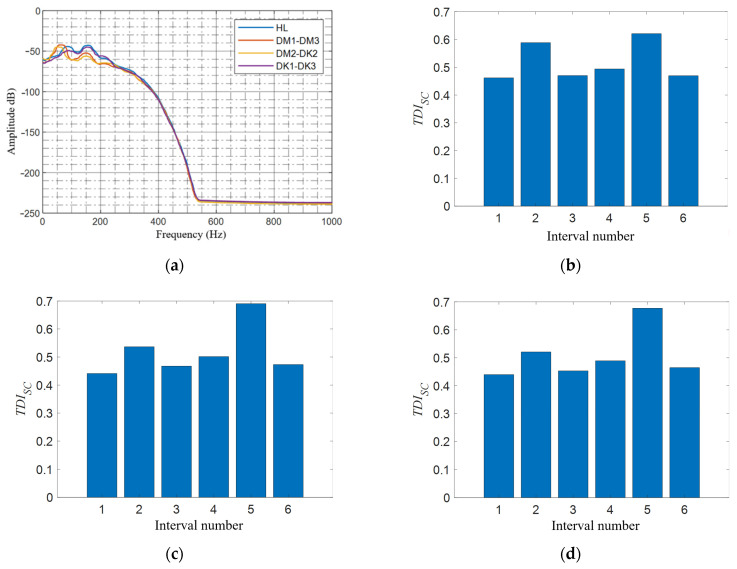
*TDI_SC_* validation in two different types of damage: (**a**) power spectrum curve of the tunnel acceleration under two different damages in the second and fifth interval; (**b**) *TDI_SC_* at additional mass damage in the second and fifth interval; (**c**) *TDI_SC_* at additional mass damage in the second interval and stiffness damage in the fifth interval; and (**d**) *TDI_SC_* at stiffness damage in the second and fifth interval.

When calculating the two damages, use groups 8 to 10 in Table 4, that is, two damage conditions with three different combinations. Figure 19a shows the tunnel acceleration power spectrum curves of two different damage and healthy conditions. The main energy distribution of each curve is mainly in the range of [0, 250] Hz, and the differences corresponding to the health and damage curves are also distributed in the range of [0, 250] Hz. Therefore, when calculating the *TDI_SC_* indicator, the calculation parameters are the same as in Section 5.2, and the result is shown in Figure 19b–d. The two maximum values of *TDI_SC_* in the figure are in the second and fifth intervals, respectively, indicating that the *TDI_SC_* index is basically effective for the scenario of two damages preset in the experiment, despite the presence of various noise interferences. This means that the *TDI_SC_* algorithm has superior multi-damage recognition capabilities.

### 5.4. Influence of Vehicle Speed

**Figure 20:**
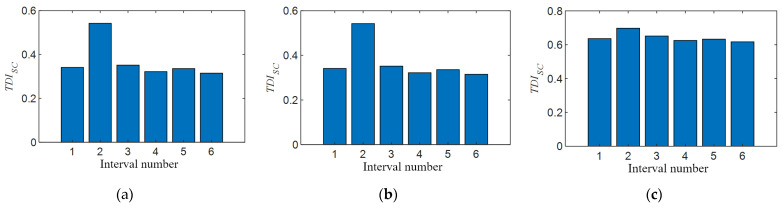
*TDI_SC_* validation at different vehicle speeds: (**a**) *TDI_SC_* of stiffness damage in the second interval when vehicle speed is 0.251 m/s; (**b**) *TDI_SC_* of stiffness damage in the second interval when vehicle speed is 0.503 m/s; and (**c**) *TDI_SC_* of stiffness damage in the second interval when vehicle speed is 0.741 m/s.

In Section 4.2.1, on the premise of no prior experience, the width of the positioning interval is determined by the size of the velocity, and the rationality of this assumption is verified by numerical calculation. This section discusses the influence of different vehicle speeds on the performance of the *TDI_SC_* index when the size of positioning interval is fixed. The data of group 17 to group 20 in addition to group 1 and group 6 in Table 4 are used for calculation. The train speeds corresponding to the converted similarity ratios are 18.07 km/h, 36.22 km/h, and 53.35 km/h, respectively. When calculating the *TDI_SC_* index, the calculation parameters are the same as in Section 5.2, and the result is shown in Figure 20a–c. In these pictures, the *TDI_SC_* value of the second interval is the largest, indicating that the *TDI_SC_* index can effectively locate to the preset damage interval under the three different vehicle speeds set in the experiment. Obviously, when the vehicle speed is lower, the *TDI_SC_* bar in the preset damage zone is more prominent in Figure 20a, which indicates that the smaller the disturbance under lower vehicle speed, the more easily it and the location of the damage are identified. Conversely, when the train speed is high, the *TDI_SC_* values of other non-damaged sections are also relatively large in Figure 20c, which almost interferes with the determination of damage location. On the one hand, this may be because when the interval is fixed, the faster the vehicle speed is, the less data can be used to calculate the interval, leading to more susceptibility to interference when calculating *TDI_SC_* values. On the other hand, the faster the speed is, the more difficult it is to ensure the consistency of test conditions before and after damage, and the more likely it is to produce noise interference.

### 5.5. Influence of Vehicle Mass

**Figure 21:**
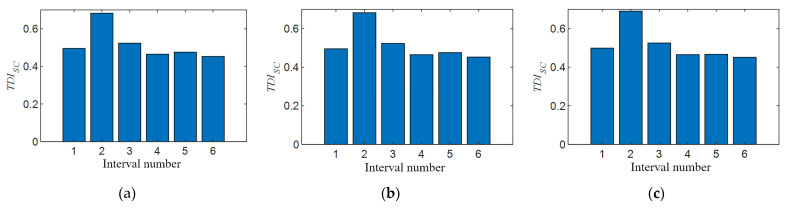
*TDI_SC_* validation at different vehicle masses: (**a**) *TDI_SC_* of stiffness damage in the second interval when load is 8 counterweight blocks; (**b**) *TDI_SC_* of stiffness damage in the second interval when load is 12 counterweight blocks; and (**c**) *TDI_SC_* of stiffness damage in the second interval when load is 16 counterweight blocks.

In this section, we study the impact of train weight on the *TDI_SC_* recognition effect, and this goal is achieved by adding different numbers of counterweights to the test vehicle. Groups 11 to 16 in Table 4 are adopted to add 8, 12, and 16 counterweight blocks to the trolley, respectively, divided into three levels to simulate the situation of light load, normal load, and overload of the train. When calculating the *TDI_SC_* index, the calculation parameters are the same as in Section 5.2, and the result is shown in Figure 21a–c. In these pictures, the *TDI_SC_* value of the second interval is the largest, indicating that the *TDI_SC_* index can also effectively locate to the preset damage interval with the different counterweights loaded on the test vehicle. In addition, with the increase of the vehicle’s load, the *TDI_SC_* index of the damage region increases slightly, which has no obvious enhancement effect on the determination of damage identification. This indicates that the change of vehicle body mass has little influence on the *TDI_SC_* index.

## 6. Conclusions

This paper presents a new method for locating the damage of a tunnel structure with a vehicle-mounted accelerometer. It locates the damage location of the tunnel structure by comparing the changes in the damage index of the vehicle body acceleration response measured before and after the tunnel damage. This is a low-cost damage location method that can be used as a useful supplement to traditional static monitoring of tunnel structures. The specific research conclusions include the following.

Firstly, a one-dimensional governing equation of vehicle–tunnel coupling vibration and its analytical solution are derived. Through the change of tunnel parameters in the control equation, the damage of the tunnel can be simulated, and the change caused by the tunnel damage can be reflected from the analytical expression of the train vibration response, so as to give the theoretical basis of this article.

Secondly, the *TDI_SC_* algorithm for tunnel structure damage location is proposed. The *TDI_SC_* algorithm divides the tunnel into equal width damage intervals according to the speed and calculates and compares the *TDI_SC_* index of each interval to realize the location of tunnel damage. The study of the refined 3D numerical simulation and scale model experiment proves that this index can not only identify the single stiffness, void, and additional mass damage of the tunnel structure, but can also identify two different types of combined damage. For the above conditions, *TDI_SC_* can recognize the damage degree even if it is only 3%. The noise test also proves that the *TDI_SC_* is still effective under the interference of white noise less than 3%, indicating that it has good robustness.

Finally, the analysis of the influencing factors of *TDI_SC_* shows that the vehicle speed has a great influence on *TDI_SC_*, and it is believed that a slow speed is conducive to *TDI_SC_* positioning damage, while the train load has little influence on *TDI_SC_,* as long as the train load remains basically the same before and after the tunnel damage.

It should be noted that the consistency of the train’s position before and after the tunnel damage is also an important factor that affects the *TDI_SC_*. This problem does not exist in the numerical simulation of this article. The model experiment avoids the influence of this factor by synchronizing the data of acceleration and vehicle position. In real applications, this factor may need to be considered for further research.

## Figures and Tables

**Table 1:** Calculation parameters of 3D tunnel and soil model.

Parameter NAME	Parameter Value	Unit of Parameter
Outer diameter of the tunnel model	6.6	m
Inner diameter of the tunnel model	5.9	m
Buried depth of the tunnel model	14.7	m
Width of each tunnel segment	1.2	m
Elastic modulus of the segment concrete (Label C55)	34,500	MPa
Elastic modulus of the track bed concrete (Label C35)	31,500	MPa
Poisson’s ratio of the segment concrete	0.2	-
Poisson’s ratio of the track bed concrete	0.2	-
Density of the segment concrete	2500	Kg/m^3^
Density of the track bed concrete	2500	Kg/m^3^
The length of soil around the tunnel model	60	m
The width of the soil around the tunnel model	60	m
The height of soil around the tunnel model	42	m

**Table 2:** Soil parameters of the tunnel model.

Soil Layer Number ^1^	Soil Name	Thickness (m)	Gravity Density (KN/m^3^)	Dynamic Modulus (MPa)	Poisson Ratio	Damping Ratio
①	Miscellaneous fill	1.7	18	30	0.3	0.03
②3	Gray clay silt	8	18.6	32.16	0.29	0.03
③	Muddy silty clay	12	18	20.16	0.29	0.03
④	Gray mucky clay	8	17.1	25.62	0.31	0.03
⑤1	Grey clay	5	17.3	39.48	0.3	0.03
⑤2	Gray sandy silt	8	17.8	73.56	0.28	0.03

^1^ The soil layer number named according to the geotechnical engineering investigation code: the number inside the circle indicates the number of the main layer and the number outside the circle indicates the number of the sub-layer.

**Table 3:** Similarity ratio of tunnel test model.

Category	Tunnel Diameter (m)	Elasticity Modulus (MPa)	Density (Kg/m^3^)	Tunnel Wall Thickness (m)
Prototype	6.6	34,500	2500	0.35
Test model	0.33	1725	900	0.0175
Similarity ratio	20	20	2.78	20

**Table 4:** Setting of experimental conditions.

Serial Number	Purpose of Analysis	Damage Type	Preset Damage Interval	Damage Level	Vehicle Speed	Number of Counterweights
1	Single damage verification	Health	/	/	0.503 m/s	12
2		Additional mass damage	2	1	0.503 m/s	12
3		Additional mass damage	2	2	0.503 m/s	12
4		Additional mass damage	2	3	0.503 m/s	12
5		Stiffness damage	2	1	0.503 m/s	12
6		Stiffness damage	2	2	0.503 m/s	12
7		Stiffness damage	2	3	0.503 m/s	12
8	Two damage verification	Two additional mass damage	2, 5	1, 3	0.503 m/s	12
9		Additional mass and stiffness damage	2, 5	2, 2	0.503 m/s	12
10		Two stiffness damage	2, 5	1, 3	0.503 m/s	12
11	Influence of train weight	Health	/	/	0.503 m/s	8
12		Health	/	/	0.503 m/s	12
13		Health	/	/	0.503 m/s	16
14		Stiffness damage	2	3	0.503 m/s	8
15		Stiffness damage	2	3	0.503 m/s	12
16		Stiffness damage	2	3	0.503 m/s	16
17	Influence of train speed	Health	/	/	0.251 m/s	12
18		Health	/	/	0.741 m/s	12
19		Stiffness damage	2	3	0.251 m/s	12
20		Stiffness damage	2	3	0.741 m/s	12
